# Primary cilia-dependent signaling is involved in regulating mesenchymal stem cell proliferation and pluripotency maintenance

**DOI:** 10.1007/s10735-020-09876-7

**Published:** 2020-05-12

**Authors:** Zhourui Ma, Mingde Qin, Hansi Liang, Ruihua Chen, Shizhong Cai, Zhijian Huang, Guangping Tai

**Affiliations:** 1 Department of Burns and Plastic Surgery, Children’s Hospital of Soochow University, Suzhou, Jiangsu Province China; 2 Key Lab of Biofabrication of Anhui Higher Education Institution, Centre for Advanced Biofabrication, Hefei University, Hefei, Anhui Province China; 3 Department of Immunology, School of Biology and Basic Medical Sciences, Soochow University, Suzhou, Jiangsu Province China; 4 Jiangsu Key Laboratory of Clinical Immunology, The First Affiliated Hospital of Soochow University, Suzhou, Jiangsu Province China; 5 Jiangsu Key Laboratory of Gastrointestinal Tumor Immunology, The First Affiliated Hospital of Soochow University, Suzhou, Jiangsu Province China; 6 Department of Child and Adolescent Healthcare, Children’s Hospital of Soochow University, Suzhou, Jiangsu Province China

**Keywords:** Primary cilia, Mesenchymal stem cells, IFT172, KIF3A, Proliferation, Cell cycle

## Abstract

Using a large-scale quantitative mesenchymal stem cells (MSCs) membrane proteomics analysis, we identified a large group of ciliary proteins in the MSCs membrane fraction, which prompted us to examine the cilia, intricate organelles that were originally discovered approximately 100 years ago. Here we characterize their primary structure and function in MSCs. We first characterized the primary cilia on undifferentiated human MSCs by immunostaining and verified these observation with scanning and 3D electronic microscopy. To investigate the function of the primary cilia of the MSCs we induced loss of function by means of siRNA knockdown (targeted to two known ciliary proteins: IFT172 and KIF3A). After either of these two proteins was knocked down by the respective siRNA, the MSCs showed fewer and shortened primary cilia. The MSCs proliferation assays showed increased cell proliferative activity under confluent conditions after the siRNA knockdown of IFT172 or KIF3A; among these MSCs, the proportion in S phase was increased in the IFT172 siRNA knockdown group. The expression of stem cell markers on the MSCs, namely, Oct4, Nanog and Sox2, were downregulated after the siRNA-induced knockdown of IFT172 or KIF3A, and the gene expression upregulation of ectoderm lineage markers was notable. Furthermore, manipulation of the primary cilia-dependent Shh pathway, using the Shh activator SAG (smoothened agonist), upregulated the gene expression of pluripotency markers, including Nanog and Oct4, and transcriptional target genes in the Shh pathway. This study confirms that MSCs have primary cilia and provides evidence that primary cilia-dependent signaling pathways play functional roles in MSCs proliferation and stemness maintenance.

**Electronic supplementary material:**

The online version of this article (10.1007/s10735-020-09876-7) contains supplementary material, which is available to authorized users.

## Introduction

Mesenchymal stromal/stem cells (MSCs) constitute a population of stromal cells in the bone marrow capable of differentiating into multiple lineages. MSCs are attractive candidates for tissue repair because of their ability to extensively proliferative in culture while retaining their multilineage differentiation potential (Xin et al. 2016). Accumulated evidence indicates that MSCs and derived progenitors are widely involved in tissue and organ regeneration. MSCs are also an excellent in vitro models for understanding complex signaling pathways that regulate pluripotency in stem cell biology (Vining and Mooney 2017); for example, the MSCs response to niche environmental factors provides important data. Over the past decade, we began to learn more about specific signaling pathways, such as the growth factor of BMPs (bone morphogen proteins), FGF2 (fibroblast growth factor 2), PDGF (platelet-derived growth factor) and transforming growth factor pathways, which are involved in modulating MSCs pluripotency and proliferation. However, we know little about how MSCs maintain their stem cell status within their niches, how MSCs sense microenvironmental niche factors (such as those of the ECM, extracellular matrix, and stiffness), how stem cells process and interpret these complex “niche” factors and signals in situ and adapt their behavior accordingly to regulate MSCs stemness, proliferation and regeneration (Kitadate et al. 2019). Compelling evidence is emerging that stem cells are able to integrate these complex “niche” signals, including chemical and physical cues provided by cell adhesion substrates, such as rigidity, topography and mechanical force signals (Kitadate et al. 2019; Yang et al. 2017). Understanding the details of these mechanisms will pave the way for experimentally manipulating niche microenvironments to maintain stem cell renewal capacity and control the differentiation and MSCs lineage specifications (Ouspenskaia et al. 2016; Haas et al. 2018).

Primary cilia emerged as cell signaling hubs for cell sensing, processing, and integrating external signals and cues on vertebrate mammalian cells (Petridou et al. 2017; Spasic and Jacobs 2017). Primary cilia are present on almost all vertebrate cells and consist of a 9 + 0 axonemal arrangement. It is estimated that more than 3000 genes encode proteins either localized to cilia or essential for cilium assembly or function. The importance of primary cilia in both health and disease reflects the roles of these organelles and their IFT system in processing, mediating and transducing intercellular chemical, physical and biological signals (Zhang et al. 2018). Primary cilia play key roles in sensing and integrating matrix factors (e.g., in the ECM) and participate in developmental patterning and morphogenic gradient sensing (Reiter and Leroux 2017; Johnson et al. 2018). Defects in primary cilia can lead to a wide spectrum of morphological abnormalities (human ciliopathies), including obesity, retinal degeneration and brain disease (Basten and Giles 2013). However, the roles of primary cilia in MSCs is largely unknown.

Following a large-scale quantitative proteomics analysis of MSCs membranes, we identified a large group of ciliary proteins in the MSCs membrane fraction, which prompted us to characterize the primary cilia on MSCs. In this study, we provide the first morphological evidence of and empirical data on the function of the primary cilia of the MSCs by knocking down two representative ciliary proteins, IFT172 and KIF3A, with siRNAs and manipulating a primary cilia-dependent signal pathways with a chemical activator. Our results demonstrate that in vitro cultured human mesenchymal stem cells (MSCs) process primary cilia under confluent culture conditions. Primary cilia are involved in the regulation of MSCs cell proliferation, cell cycle regulation, and MSCs pluripotency maintenance.

## Results

### Identification of primary cilia proteins from MSCs through a quantitative membrane proteomic study

This study is based on a previous quantitative membrane proteomics study (Lai et al. 2011). Three cell types from different donors were used: human bone marrow MSCs, human umbilical cord perivascular cells (HUCPVCs) and human dermal fibroblasts (HDFs). The fractionated membrane proteins were purified, and a total of eight samples were used (three samples of MSCs and HUCPVC cells and two samples of HDFs, all from different donors). Each sample was labeled separately with an 8-Plex iTRAQ labeling kit, and pooled samples were subjected to a single mass spectrum analysis with an AB SCIEX TripleTOF® 5600 + System to guarantee identical conditions and to minimize variability This analysis led to the identification of 5300 membrane proteins. A panel of 153 proteins enriched in both the MSCs and HUCPVC samples was subjected to a separate stem cell marker discovery study (Lai et al. 2011). In this study, we focused on a group of unique ciliary proteins identified through a quantitative membrane proteomics analysis.

We identified a list of ciliary proteins expressed on the MSCs membrane, including three in the KIF3 family (KIF3A, KIF3B, and KAP3), seven in the IFT family (IFT172, IFT88, IFT81, IFT74, IFT57, IFT52 and IFT27) encoded by IFT genes, and others, including OFD1 and retrograde IFT member DYNC 2H1; IFTA complex (IFT140) proteins; ciliary transition zone proteins, namely, Tmem67, CC2D2A, and Tctex-1; and cilia base protein OFD1 (supplementary data Table S1).

We were intrigued upon finding that these structural ciliary proteins were expressed on MSCs, as little is known about the role of the primary cilia of the MSCs.

### Three-dimensional view of primary cilia on MSCs

**Fig. 1:**
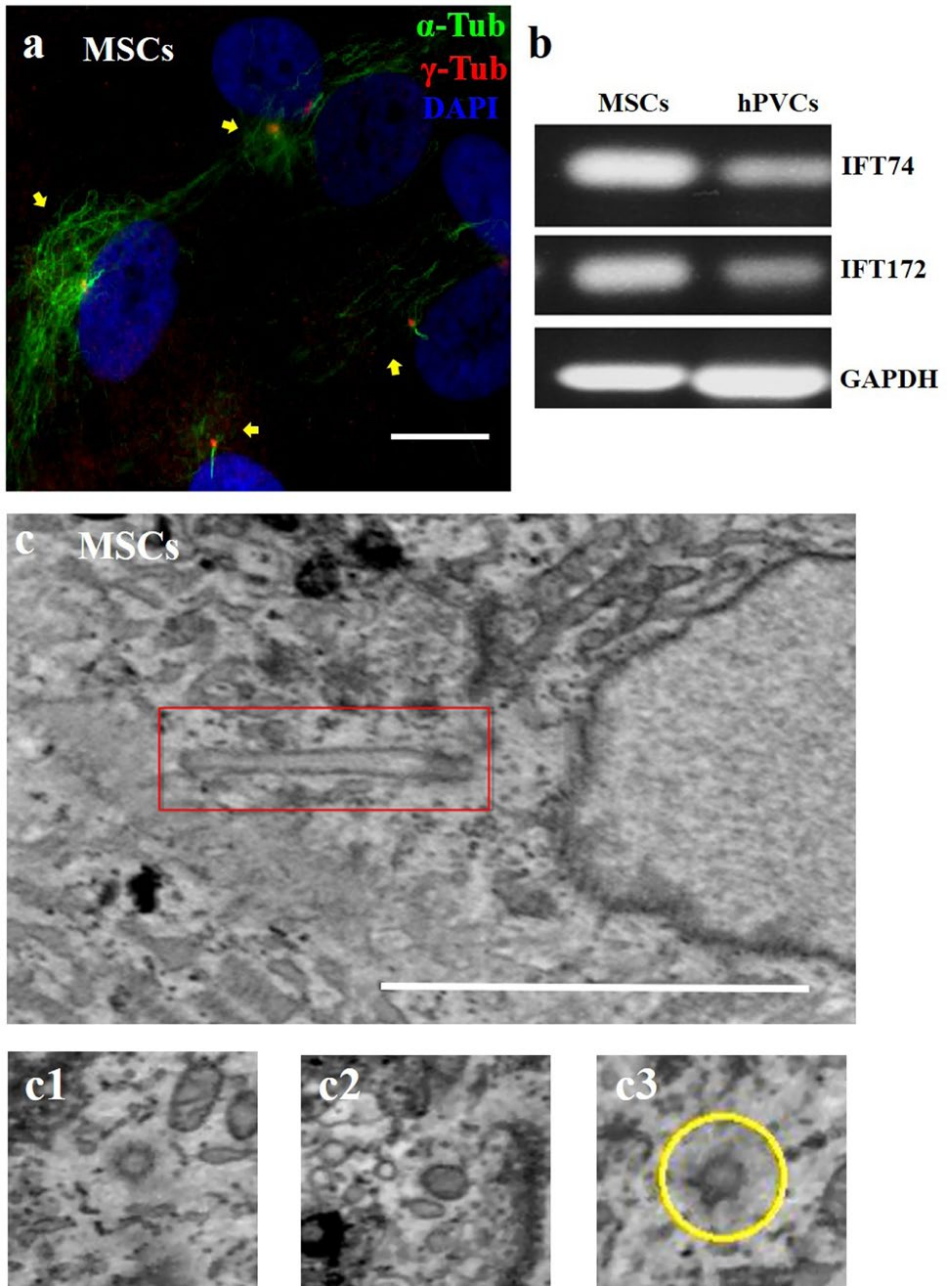
MSCs have primary cilia. RNA was isolated from MSCs and hPVCs. Primary cilia were stained with acetylated α-tubulin (green) and **γ-**tubulin (red) in cultured confluent MSCs at 100 µM (**a**). Gene expression of ciliary proteins IFT74 and IFT172 was measured by RT-PCR (**b**). Ultrastructure of primary cilia was characterized by transmission electron microscopy (**c**). Three-dimensional electronic microscope view of primary cilia (c1, c2, c3). Scale bar, 500 nm in (**a**), is the same bar as that shown in (**c**)

To validate the proteomics analysis findings, we performed semi-quantitative RT-PCR (reverse transcription-polymerase chain reaction) analyses, and more importantly, we confirmed primary cilia on MSCs by morphological analysis. MSCs were cultured to confluence on gelatin-coated coverslip. We used immunostaining of acetylated α-tubulin and **γ**-tubulin to visualize the primary cilia (Fig. 1a). The expression levels of two representative genes, *IFT74 and IFT172,* and their respective mRNAs were measured, confirming that their encoded proteins were expressed on the MSCs (Fig. 1b). Primary cilia were found on 10–14% of the MSCs under confluent culture conditions (5 days after confluence was reached). As no previous work has demonstrated the ultrastructure of primary cilia on human MSCs, we performed an electronic microscopy imaging analysis using two different electronic microscopic techniques: scanning electron microscopy and 3D transmission electron microscopy (TEM). Each MSCs cell harbored a single cilium (Fig. 1c). Images from 3D transmission electron microscopy showed the full length of the primary cilium, and its base body was visualized. The primary cilium was deeply rooted within the cytoplasm, close to the nucleus, and emerged at the cell surface (Fig. 1c1–c3).

### Knocking down IFT172 impairs MSCs ciliogenesis

**Fig. 2:**
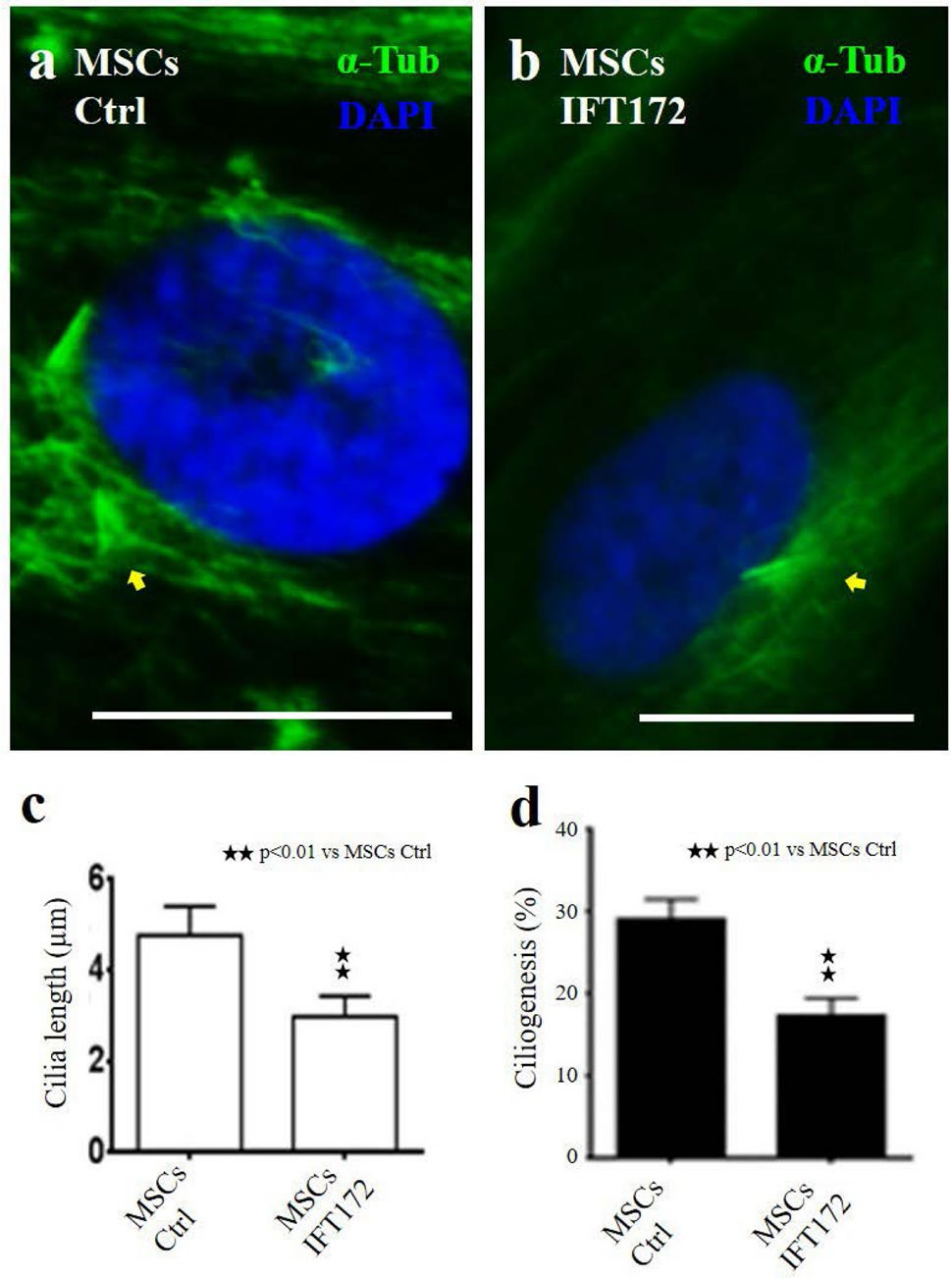
Knocking down IFT172 impairs MSCs ciliogenesis. Primary cilia were visualized by the combined staining of α-tubulin and **γ**-tubulin (**a**, **b**). The length of the cilia was measured by ImageJ software (**c**). The stained primary cilia were counted and reported as the number per 1000 nuclei, and the ratio was calculated (**d**). Scale bars: 20 µM

Having confirmed the presence of primary cilia on MSCs in culture, we next explored the effects of knocking down one key ciliary protein by siRNA as a means of introducing loss of function of this ciliary protein. We chose to downregulate key ciliary protein IFT172. After three repeat siRNA knockdown treatments on three consecutive days, most control MSCs presented with a single primary cilium per cell, and a few IFT172 siRNA knocked down MSCs presented with two cilia on one cell. One prominent feature of the IFT172 siRNA knocked down MSCs was shorter cilia, with the average length of the cilia being 2.5 micrometres in the IFT172 siRNA knocked down MSCs compared to cilia of 4.5 micrometres in the control MSCs (Fig. 2a–c). Quantification of the primary cilia indicated that the number of MSCs cells with a primary cilium was lower in the siRNA knocked down MSCs (15% in the siRNA knocked down MSCs population) than it was in control MSCs (30% of the MSCs population had a primary cilium) (Fig. 2d). qPCR confirmed that the IFT172 siRNA-induced knockdown was successful (the knockdown efficiency was 99% at 48 h) (Fig. S1).

### IFT172 siRNA-induced knockdown enhances cell proliferation activity

**Fig. 3:**
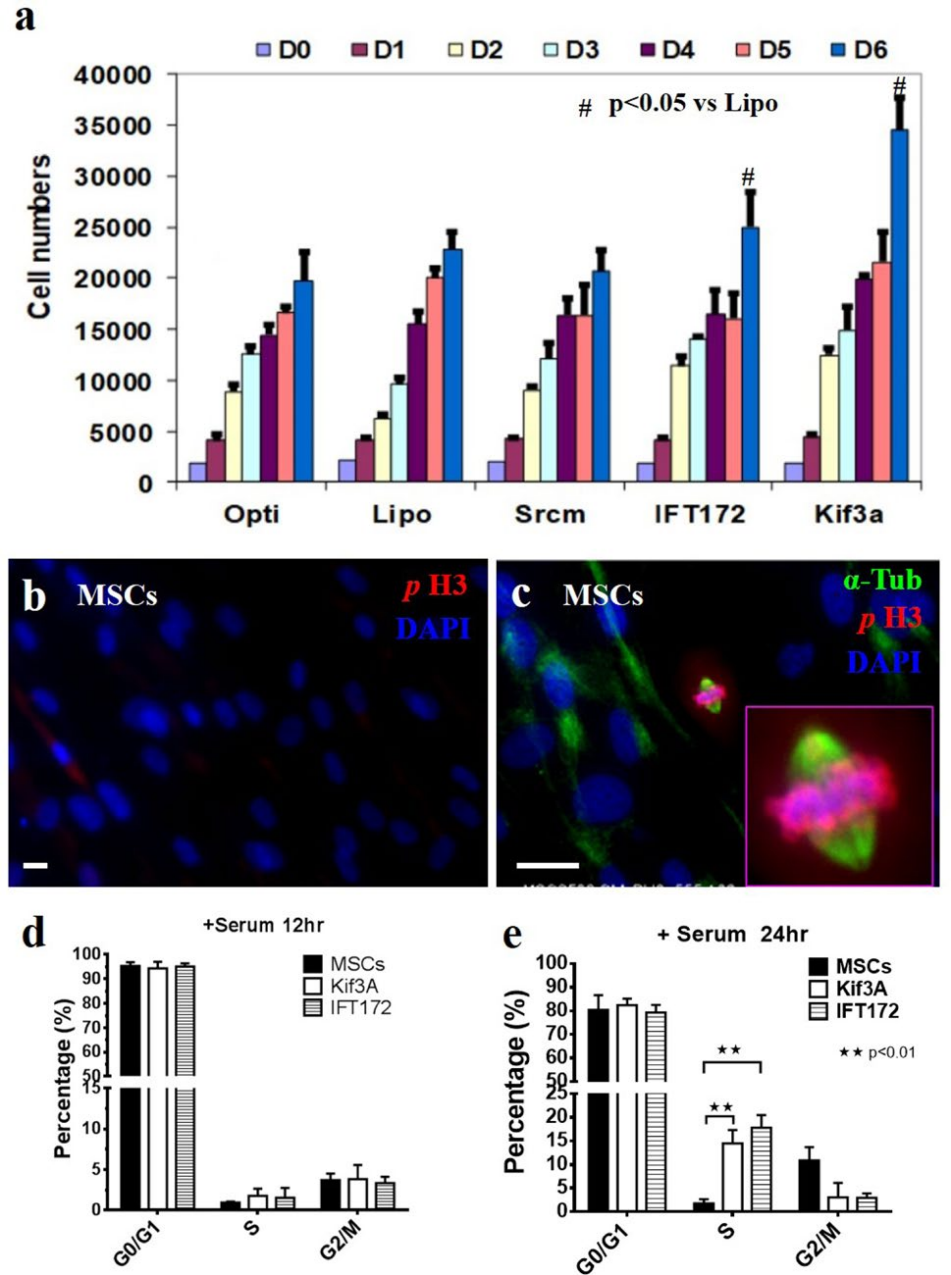
IFT172 and KIF3A siRNA knockdown promotes cell proliferation and cell cycling in confluent MSCs. Mesenchymal stem cells were cultured for 6 days and treated with siRNA on D1 D3 and D5. MSCs were collected on D0, D1, D2, D3, D4, D5 and D6, and the DNA content of proliferating MSCs was quantified by CyQUANT. Mitotic MSCs under confluent conditions (150,000 cells per well in 12 wells for 6 days) were stained for phosphorylated histone H3. For the cell cycle analysis, MSCs were cultured to confluence (at a density of 150,000 per well in a 12-well plate and cultured for 6 days, and treated with siRNA three times on D1 D3 and D5). MSCs were serum starved for 24 h and then passaged at a 1:3 ratio in standard medium containing serum. MSCs were collected and fixed at 0 h, 12 h and 24 h. MSCs were stained with propidium iodide, and the cell cycle was analyzed with a Beckman Coulter Cyan ADP flow cytometer

The length of primary cilia and cell proliferation are interconnected. Therefore, we investigated the extent wo which MSCs proliferation was regulated by primary cilia by using siRNA to knock down two ciliary proteins: IFT172 and motor protein KIF3A. Mesenchymal stem cells were cultured for 6 days and treated with siRNA on days 1, 3 and 5. The MSCs were observed on days 1, 2, 3, 4, 5 and 6. MSCs proliferation was quantified by DNA content. siRNA-induced knockdown resulted in no significant differences in cell proliferation from day 1 to day 4, and number of cells (measured by DNA content) was significantly higher in both knockdown groups (IFT172 or KIF3A siRNA knockdown) in compared to that of the control group on day 6 (P < 0.05, IFT172 siRNA knockdown vs control groups; P < 0.05 KIF3A vs control groups) (Fig. 3a). These results were unexpected, as both KIF3A and IFT172 siRNA knocked down MSCs showed higher proliferation (DNA content) than the control MSCs when the MSCs had reached confluency, after 6 days of cell culture. To verify the cell proliferation status, MSCs were cultured for 6 days and treated with three repeat siRNA knockouts (on days 1, 3 and 5). The MSCs were fixed on day 6 and stained for phosphorylated histone H3, a mitosis-specific marker. There was no phosphorylated histone H3 staining in the confluent control MSCs; however, there were phosphorylated histone H3-positive cells among the IFT172-knockdown MSCs population (Fig. 3b–c).

Because of technical difficulties in generating suitable individual MSCs from the confluent MSCs culture, we verified the cell cycle status of the confluent MSCs after IFT172 siRNA knockdown. The cell cycle analysis was performed using flow cytometry. As MSCs produce a rich extracellular matrix in confluent culture, an alternative approach was used. The MSCs were first cultured to confluence for 6 days with IFT172 siRNA knockdown repeated on days 1, 3 and 5. Then, the MSCs were serum starved for 24 h to synchronize the cell cycle, and the MSCs were then passaged and seeded onto new plates at a 1:3 ratio in standard medium containing serum. MSCs samples were taken after 24 h, fixed, and subjected to flow cytometric analysis. A higher proportion of MSCs in the IFT172 siRNA knockdown group were in the S phase after 24 h of culture, which confirmed that the IFT172 siRNA knockdown altered the cell cycle status (Fig. 3d–e).

### IFT172 and KIF3A siRNA knockdown downregulates pluripotent gene expression

**Fig. 4:**
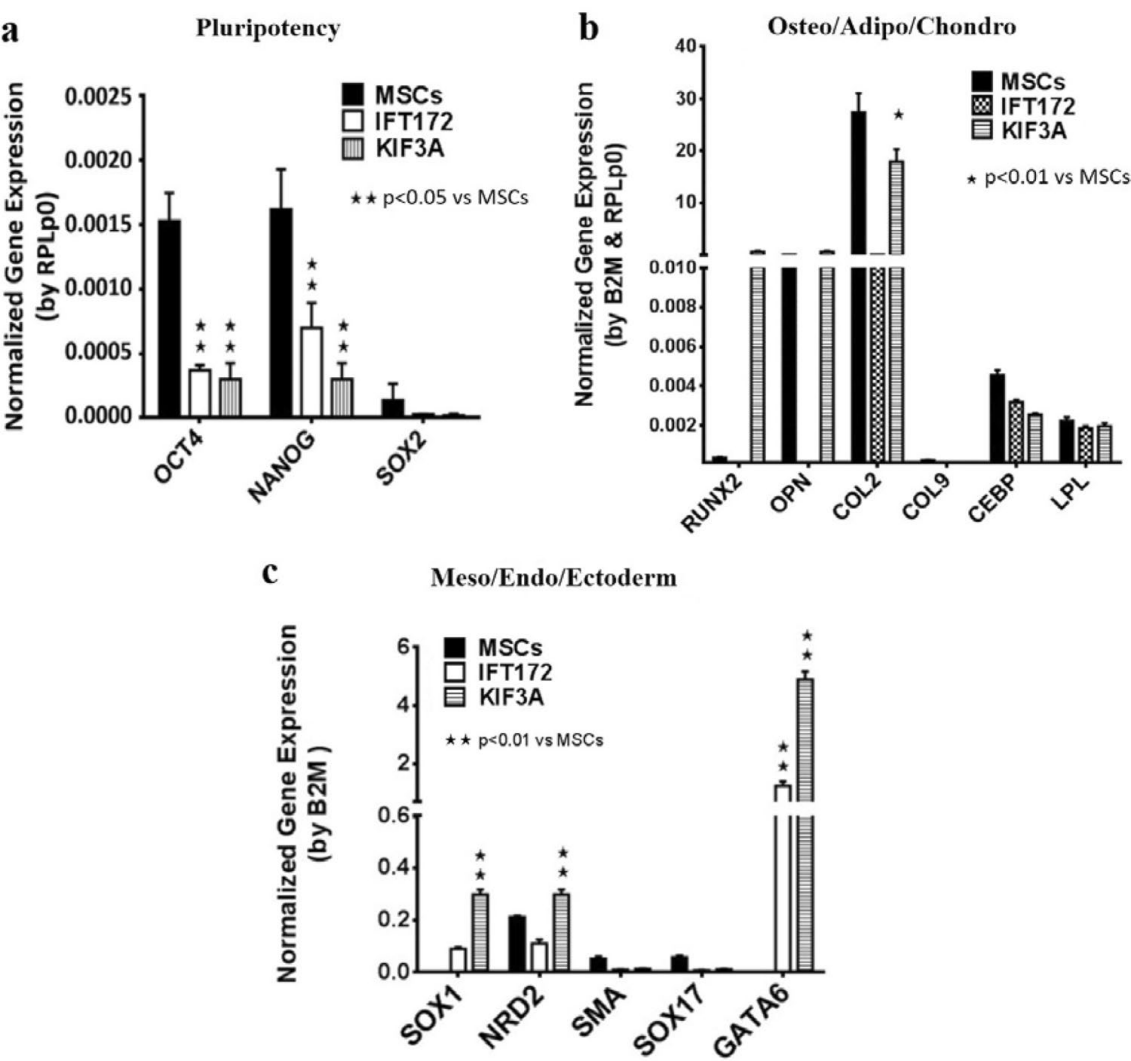
IFT172 and KIF3A siRNA knockdown initiates MSCs differentiation and downregulates pluripotency-related gene expression. Mesenchymal stem cells were cultured in MesenPRO medium for 6 days and treated with siRNA on D1 D3 and D5. qPCR was carried out to quantify the gene expression of the pluripotent transcription factors and endoderm, ectoderm and mesoderm lineage markers. Data on the MSCs from two different MSCs donors are presented from three experiments

Next, we asked whether primary cilia are involved in MSCs pluripotency maintenance. We examined the gene expression of pluripotent genes and three lineage marker genes after siRNA knockdown. MSCs were cultured in standard maintenance medium for 6 days with siRNA knockdown repeated on days 1, 3 and 5. Quantitative qPCR was used to quantify the gene expression. The expression of pluripotent marker gene, namely, Oct4, Nanog and Sox2, was decreased significantly after IFT172 or KIF3A siRNA knockdown (Fig. 4a). Differentiation markers, namely, osteogenic lineage markers (Runx2 and Osteopontin), were upregulated after IFT172 siRNA knockdown and increased significantly after siRNA knockdown of IFT172 or KIF3A (Fig. 4b). Ectoderm markers, namely, Sox1, NRD2 and GATA6, were significantly upregulated in both the IFT172 siRNA knockdown group and the KIF3A siRNA knockdown group (Fig. 4c). The efficiency of the siRNA knockdown was validated by quantitative qPCR, which showed that the knockdown efficiency was 95% and 94% for IFT172 and KIF3A, respectively (Fig. S2).

### Shh pathway activation upregulates pluripotent gene expression

**Fig. 5:**
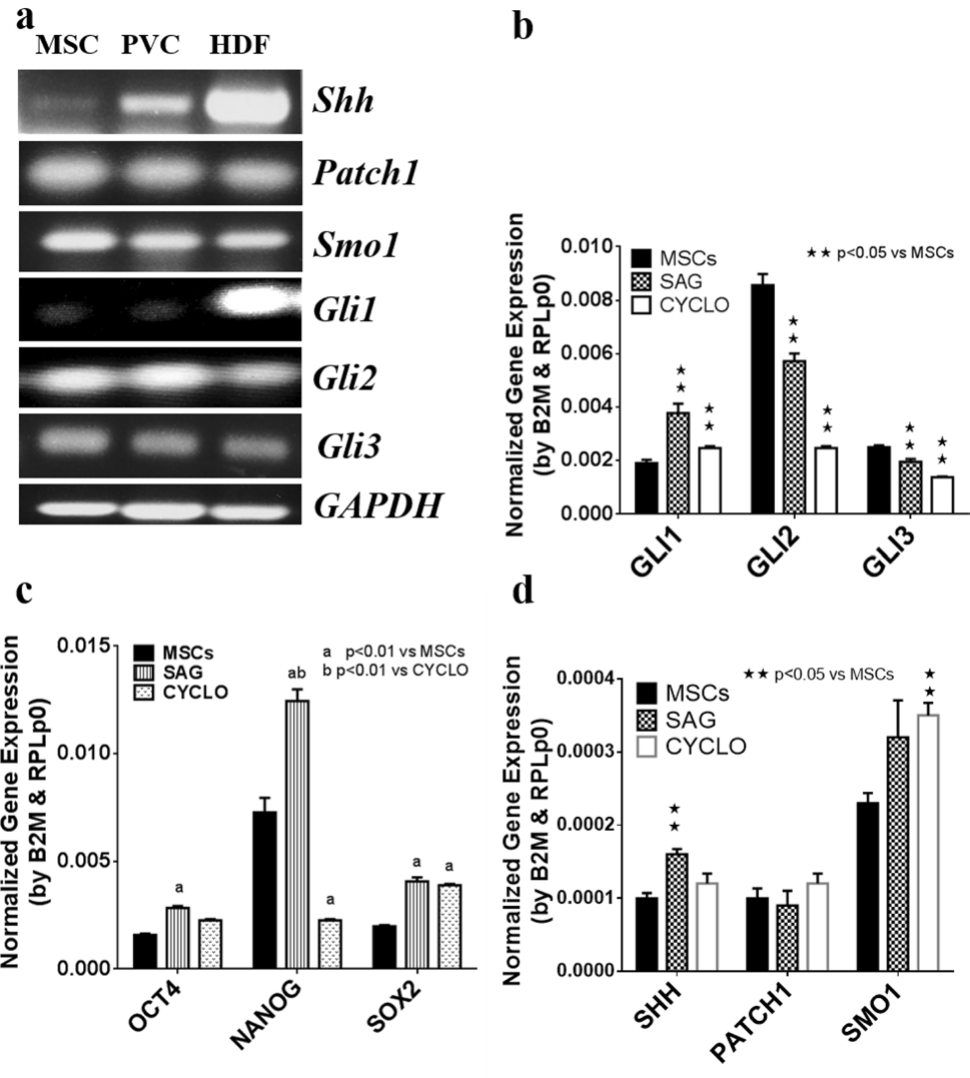
SAG activates the shh pathway and upregulates pluripotent gene expression in the MSCs. Mesenchymal stem cells were treated with 100 nM SAG and 2.5 µM cyclopamine for 48 h, mRNA was isolated, and gene expression was quantified by qPCR

The extent to which primary cilium-mediated signaling pathways are involved in MSCs pluripotency maintenance remains unclear. The Shh (Sonic hedgehog) signaling pathway is an established primary cilium-dependent pathway. We sought to examine the potential role of Shh in MSCs pluripotency maintenance. The results from the qPCR analysis confirmed the gene expression of Shh pathway components in the MSCs, hPVCs and HDFs. These genes included Shh ligands, two receptors, Patched 1 and Smo1, and three transcriptional target genes (Gli1, Gli2 and Gli3) (Fig. 5a). Pluripotent genes (Oct4, Nanog and Sox2) were decreased after IFT172 or KIF3A siRNA knockdown (Fig. 5c). The sonic hedgehog (Shh) pathway activator SAG (a cell-permeable smoothened agonist) was used to treat the MSCs for 48 h. Activation of shh downstream transcriptional target genes (Gli1 Gli2 and Gli3) was evident (Fig. 5b). Quantitative qPCR analysis showed that the gene expression of pluripotency markers, including nanog and Oct4, was increased significantly (p < 0.05, Fig. 5c), correlating with the upregulation of Shh pathway transcriptional targets, including Gli1 Gli2 and Gli3, after 48 h of treatment.

## Discussion

This study unveiled mesenchymal stem cell processes in primary cilia. Knocking down ciliary proteins KIF3A or IFT172 interrupted primary ciliogenesis and altered fundamental cell functions, such as proliferation activity and cell cycle regulation. Knocking down IFT172 impaired the balance of stem status maintenance and initiated cell differentiation. Manipulation of primary cilium-mediated signals, such as the activation of the shh signaling pathway, altered the stem cell status in terms of self-renewal capacity and pluripotent gene expression.

The advancement of proteomics techniques has enabled large-scale protein identification. An 8-Plex ITRAQ labeling method was the best means to label as many as eight samples separately before the samples were combined for the single mass spectrum analysis, which greatly minimized the variation and potential contamination. This analysis led to the identification of 5300 membrane proteins (Lai et al. 2011). More than 23 ciliary cells were identified in this assay, which prompted us to investigate the roles of primary cilia on MSCs.

We identified a list of ciliary proteins on MSCs membranes, including members of the KIF3 family (KIF3A, KIF3B, and KAP3), IFT family members OFD1 and DYNC 2H1, IFTA complex (IFT140) and ciliary transition zone proteins (Table S1). The number of identified ciliary proteins was lower than that for the ciliary proteomics analysis using epithelial cells, which reported that more than 3000 proteins were identified (Johnson et al. 2018; Frishman-levy and Izraeli 2017). The discrepancy is related to two factors: the membrane enrichment method used in the current study was different from that used for the ciliary-specific proteomics method previously reported, and the abundance of primary cilia on epithelial cells is likely greater than it is on MSCs (Holley et al. 2015). The current cell culture and the methodology used for protein enrichment were not designed for primary ciliary proteomics. Optimization is needed for future studies to identify all the ciliary proteins.

A morphological assessment is the best technique to prove the presence of primary cilia; however, reports of primary cilia have not been well established in the stem cell biology literature to date. Sreekumar (Sreekumar et al. 2018) reported that primary cilia play roles in guarding and switching the lineage commitment of MSCs but showed no morphological evidence. Gupta et al. showed low-resolution images as evidence of primary cilia immunostaining (Gupta et al. 2018). In the current study, MSCs were cultured at a high density, and 30% of the MSCs were found to have primary cilia, as evidenced by duo-antibody immunostaining of the acetylated actin and tubulin. Furthermore, in this study, two alternative electronic microscopy techniques were used to visualize the primary cilia, including three-dimensional and scanning electronic microscope methods. Advanced electronic microscopy techniques enabled us to detect the abnormal morphology of the primary cilia, similar to cells with genetic defects in a ciliary protein (Ghossoub et al. 2016; Sun et al. 2019). Nathwani (Nathwani et al. 2014) observed the morphological differences in the primary cilia of iPSCs and their parent cells. According to the literature, primary cilia were also indirectly visualized by using a green fluorescent protein fusion protein to track inner cilium components during cell migration. Kiprilov (Kiprilov et al. 2008) first provided morphological evidence by using the GFP-tracking technique to track hedgehog signaling machinery on human embryonic stem cells. However, no functional evidence of primary cilia on human embryonic stem cells was reported in this study. The roles of primary cilia in terms of stem cell biology are largely unknown.

Among the identified ciliary proteins, two ciliary proteins were selected for functional studies. IFT172 is the largest protein in complex B, and KIF3A is the critical motor protein driving the protein complex trafficking inside cilia (Kwon et al. 2010; Inglis et al. 2006). Knocking down IFT172 or KIF3A impaired primary ciliogenesis and affected the MSCs proliferation signals under confluent cell culture conditions. Morphologically, fewer primary cilia and shorter primary cilia were observed in protein-knockdown MSCs. Accordingly, abnormal MSCs proliferation activity was observed for the MSCs treated with IFT172 siRNA. These results were confirmed by the DNA content measurement and cell cycle analysis. It is intriguing that a higher percentage of the IFT172 siRNA knockdown MSCs were in the S phase after they had been transitioned from confluent culture conditions to lower density culture conditions. This result was consistent with the observation from Robert (Robert et al. 2007); IFT88 knockdown alleviated the cell cycle restriction and favored cell proliferation, IFT88 (Polaris) participated in the suppression of the cell cycle G1/S transition, and the overexpression of IFT88 promoted the arrest of HeLa cells in the G1 phase. These findings were also supported by those of another study in which IFT20 deletion (in complex B) enhanced the proliferation of kidney collecting duct cells and was accompanied by the abnormal orientation of the mitotic spindle (Delaval et al. 2011). IF172 knockdown in the MSCs consequently affected cilia-mediated signalling, which contributed to the abnormal MSCs proliferation observed for confluent conditions. siRNA knockdown of ciliary protein (IFT172 and KIF3A) compromised normal cell proliferation regulation, such as the release from a restricted cell cycle. siRNA knockdown (IFT172 and KIF3A) impaired the integrity of primary cilia, which likely affected the ciliary trafficking and signal transduction.

Primary cilia act as both mechano- and chemosensors in cell signal transduction cascades, including those of Hedgehog (Hh) and platelet-derived growth factor (PDGF) pathways, and they modulate intracellular calcium signals in most mammalian cells (Bangs and Anderson 2017; Clement et al. 2013). It has been established that the balance between stemness status and the self-renewal and differentiation capacities of MSCs is sustained by a complex intricate network of growth factors and transcription factors that orchestrate proliferation and stem cell differentiation (Kitadate et al. 2019; Yang et al. 2017). Several well-established signaling pathways are regarded as important in stem cell proliferation and MSCs maintenance, but their association with primary cilia remains unclear. Two pathways, the PDGFR and Shh pathways, have been reported to be activated through primary cilia. Transduction of PDGF signaling via PDGFR-alpha signaling has been extensively demonstrated to be dependent on fibroblast primary cilia. Schneider showed that in cells from PDGF-AA treated IFT88 mutant mice, the levels of PDGFRα were not upregulated; thus, PDGFR signal transduction was interrupted because of the faulty primary cilia (Schneider et al. 2010). It may be the case that, in adult MSCs, the PDGF signaling pathway is involved in controlling cell proliferation and stemness maintenance. This supposition is supported by the current findings showing that IFT172 and KIF3A knockdown of MSCs induced spontaneous differentiation, even in standard stem cell maintenance medium. However, the roles of primary cilia-dependent PDGF signals in stem cell biology have been largely unexplored.

The Shh signaling pathway is a well-established signaling pathway, and its signal transduction is largely dependent on intact primary cilia (Basten and Giles 2013; Bangs and Anderson 2017). Interestingly, Kiprilov (Kiprilov et al. 2008) reported that ciliary trafficking is active in embryonic stem cells, Shh stimulates Smo trafficking to the plasma membrane of embryonic stem cells and induces the expression of Shh-target genes. In this study, the siRNA knockdown of IFT172 in the MSCs downregulated pluripotent gene expression and promoted spontaneous cell differentiation. On the other hand, when a small chemical compound, the Smo agonist SAG, was used to activate the Shh pathway, stemness genes, including Oct4, Nanog, and Sox2, were upregulated, indicating the role of Shh in MSCs pluripotency maintenance. This evidence reinforces the notion that primary cilia and their dependent signal pathways play roles in fine-tuning the balance of stem cell self-renewal and stem cell differentiation (Anvarian et al. 2019; Nachury and Mick 2019; Mitchison and Valente 2017). However, questions remain regarding the extent to which primary cilia-mediated pathways are involved in the pluripotency regulation of adult and embryonic stem cells.

In conclusion, this study confirms that MSCs have primary cilia and that these cilia are involved in modulating MSCs proliferation and maintenance. This study sheds new light on the underlying mechanisms and the possible roles of primary cilia-mediated signaling in stem cell biology.

## Materials and methods

### Cell culture and reagents

Human MSCs (purchased from Lonza were derived from three different healthy donors: MSC4 Lonza Lot 6F4085, MSC1 Lonza Lot 7F3674, and MSC2 Lonza Lot 6F4085). These MSCs were cultured in MesenPRO medium (Gibco cat 12747) supplemented with 10 ml of growth medium, 5 ml of penicillin/streptomycin and 5 ml of l-glutamine (Gibco). HuPVC (purchased from Lonza) and in 10% FCS MEM (Sigma Cat M4526) with 10,000 units of penicillin/streptomycin and 5 ml of l-glutamine. Human dermal fibroblasts (purchased from Lonza) were cultured in DMEM (Cat BE12604F). Smoothened agonist SAG (Cat 566660-1MG)(Merck Calbiochem) and other compounds and reagents were obtained from Sigma Chemical (Poole, UK), unless otherwise stated.

### Membrane proteomics analysis

Plasma membranes were prepared from enriched. MSCs grown to 90% confluence and pelleted. The membranes were enriched as previously described (Lai et al. 2011). Protein samples were digested overnight with trypsin (1:10 enzyme:protein) at 37 °C before being labeled with iTRAQ reagent (Applied Biosystems). The samples were pooled prior to the MS analysis, as previously described (Lai et al. 2011).

### Immunohistochemical analysis

The cells are cultured in in MesenPRO medium, and the MSCs cells were fixed after 6 days, when they had become confluent. Primary cilia were stained with anti-acetylated α-tubulin (from Abcam ab24610) and rabbit anti-**γ**-tubulin antibody (from Abcam ab16504). Mitotic cells were stained to detect phospho-histone H3 (stain from Abcam ab5176) and subsequently with the appropriate fluorochrome-conjugated secondary antibodies (Alexa Flour 488 and Alexa Flour 568, Invitrogen). Nuclei were stained with DAPI (Invitrogen, 1:600) and counted. Slides were mounted with VECTASHIELD reagent (Vector Labs). Images were captured using a Cool Snap ES camera (Photometrics) with MetaVue software (Molecular Devices) or a Nikon digital camera and analyzed and Image J software.

### Electron microscopy analysis

Transmission electron microscopy (TEM) was used for primary cilia imaging. MSCs were fixed with 4% formaldehyde + 2.5% glutaraldehyde in 0.1 M HEPES buffer (pH 7.2) for 1 h, scraped and pelleted. The cells were postfixed with 1% osmium tetroxide + 1.5% potassium ferrocyanide in 0.1 M cacodylate buffer (pH 7.2) for 1 h, 1% thiocarbohydrazide in water for 20 min, 2% osmium tetroxide in water for 30 min, and then in 1% uranyl acetate in water overnight. The next day, the cells are stained with Walton lead aspartate (Walton, 1979) for 1 h at 60 °C. After staining, the samples were dehydrated in an ethanol series infiltrated with TAAB 812 hard grade resin and polymerized for 24 h at 60 °C. For routine TEM, sections were cut with a Reichert Ultracut ultramicrotome and observed with a FEI Tecnai 12 BioTWIN microscope at a 100 kV accelerating voltage. Images were taken with a Gatan Orius SC1000 CCD camera.

For 3D transmission electron microscopy (TEM) and serial block face SEM (SBF-SEM), the polymerized samples were trimmed and glued to aluminum pins. Sectioning of slices with a thickness of 100 nm was completed by a Gatan 3-View attachment in automatic mode. The images were taken with an FEI Quanta 250 FEG scanning electron microscope at a 3.8 kV accelerating voltage and with 0.4 Torr chamber pressure.

For SEM, MSCs cells were grown on 0.01% gelatin-coated coverslips and fixed with 4% formaldehyde + 2.5% glutaraldehyde in 0.1 M HEPES buffer (pH 7.2) for 1 h. Then, the cells were postfixed with 1% osmium tetroxide in water for 1 h. The cells were dehydrated in acetone and critical point dried. The coverslips were mounted on aluminum stubs, a conductive bridge was made with silver DAG, and a gold/palladium sputter coating was applied. The cells were observed under high vacuum conditions with a FEI Quanta 250 FEG SEM at a 5 kV accelerating voltage.

### RNA isolation and real-time PCR

RNA was isolated with TRIzol reagent (Invitrogen) followed by use of a RQ1 DNase kit (Promega) to remove potential genomic DNA contamination. Standard reverse transcription was carried out to generate cDNA.

Real-time PCR was performed on a Bio-Rad CFX touch 1000 qPCR system, and qPCR was carried out by using Bio-Rad CFX Manager software. The validated primers used are listed in Table S2. The housekeeping gene RPL13A (Curtis et al. 2010) and other validated primers were from Qiagen. The qPCR results were pooled for a gene study using Bio-Rad CFX Manager software.

### siRNAs

IFT172- and KIF3A-knockdown MSCs cells were generated using ready-to-use custom synthesized siRNA (Eurofins MWG Operon, Germany) against human IFT172 (target sequence: 5′- GCUGCUGAUCUCUCAUUACUA-3′) and KIF3A (target sequence: 5′-UCAUGUGCCUUAUCGUAACUCUA-3′) or scrambled oligonucleotides (mock), all at 50 nM, and Life Technology transfection reagent (RNAmax) according to the manufacturer’s instructions. Cells at 60% confluency were transfected. The day after the siRNA treatment, qPCR was used to verify successful knockdown.

### Cell proliferation assay

Cells (2000 MSCs/well) were plated on a 96-well plate. Triplicate wells were used for each knockdown treatment. The cells are allowed to attach overnight before the initial siRNA transfection was conducted. The final concentration of siRNA was 40 nM, which was equivalent to 4 pmol, in 96-well plates with Lipofectamine RNAiMAX (Invitrogen), and a scramble siRNA control (Ambion AM4613) and Lipofectamine-only control were included. A set of seven culture plates was used and collected daily (from day 0 to day 6). Standard plates were established by using serial triplicate wells with cell numbers ranging from 50 to 50,000 cells. siRNA transfection are carried out on days 1, 3, and 5. The cell culture plates were frozen and stored at – 70 °C before the CyQUANT analysis. Cell proliferation was measured with a CyQUANT® cell proliferation assay kit (C7026, Invitrogen). In brief, the cells in the 96-well plate were removed from the − 70 °C freezer and thawed, and then, 200 µl of mixed lysis buffer was added, and the cells were counted by a FLX-800 fluorescence microplate reader (Bio TEK Instruments Inc) at 480 nm excitation and ~ 520 nm emission. KC-Junior software was used for the measurements.

### Flow cytometry analysis

MSCs were seeded at 5 × 10^5^ cells in Falcon (T25) flasks. The cells reached confluency after 6 days in culture with three treatments of IFT172 siRNA or KIF3A siRNA (40 nM) to create knockdown cells. The MSCs were passaged into new flasks, and the cells are collected at 0, 12 and 24 h later and the cells were fixed with ice-cold 70% ethanol. The cells were stained overnight with propidium iodide and ribonuclease A for the cell cycle analysis by flow cytometry (Beckman Coulter Cyan ADP).

### Statistical analyses

The statistical analysis was performed using Student’s *t*-tests, with a p value < 0.01 indicating significance. The data are presented as the means ± standard error of the mean unless otherwise stated.

## Electronic supplementary material

Below is the link to the electronic supplementary material.

Supplementary file1 (DOCX 343 kb)
