# Supplementary material for: Primary cilia-dependent signaling is involved in regulating mesenchymal stem cell proliferation and pluripotency maintenance
Source: J Mol Histol. 2020 May 12;51(3):241–50. doi: 10.1007/s10735-020-09876-7 (PMC7253378; doi:10.1007/s10735-020-09876-7)
Supplement: Supplementary file 1 — Supplementary file1 (DOCX 343 kb) [file 10735_2020_9876_MOESM1_ESM.docx]

**Supplementary data**


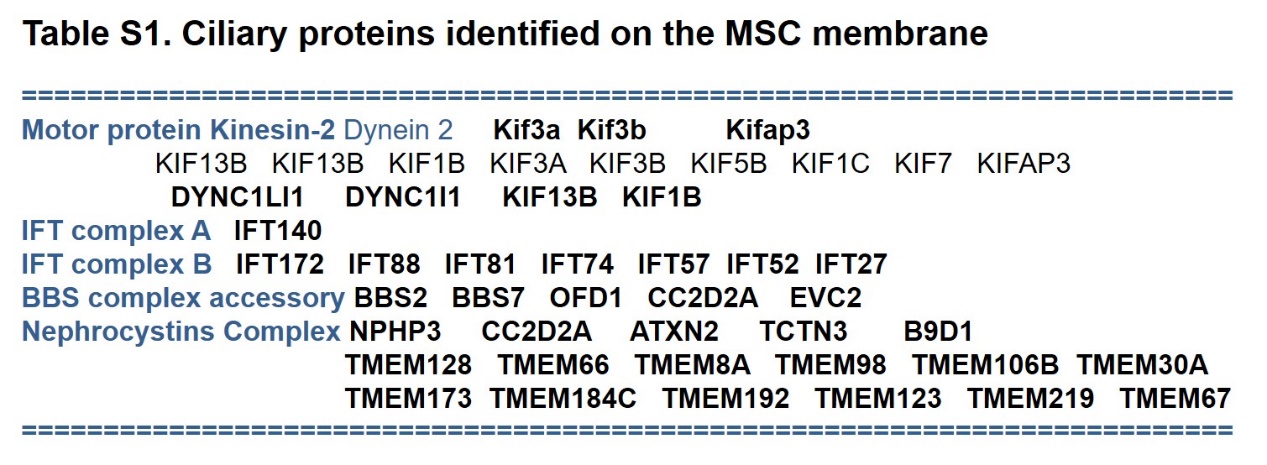


**Table S1. List of identified ciliary proteins.** Ciliary proteins expressed on the MSCs membranes as identified from the 5300 membrane proteins identified.

**Table S2. Primers used for qPCR**

Other primers, including GATA6, Brackury, SOX1, B2M, and RPLp0, from Qiagen were validated

------------------------------------------------------------------------------------------

Trans-gene Forward primer Reverse primer

------------------------------------------------------------------------------------------

C/EBP ALPHA caagagccgcgacaaggcca gcttgcgcaggcggtcattg

LPL tggccgtgtggctccagagt tcttctttggtcggcggcgg

RUNX2 cttcctgccatcaccgatgt gccagaggcagaagtcagag

Osteopontin tcacctgtgccataccagtta tggtcatggctttcgttgga

Collagen type IIA1 ggctcccagaacatcacc atgagcagggccttcttg

Collagen type IXA2 aggaatagggcggctttc aagatggccagtggagga

Oct4 gccgtatgagttctgtgg tctccttctccagcttcac

Nanog attatgcaggcaactcactt gattctttacagtcggatgc

Sox2 taagtactggcgaaccatct aaattaccaacggtgtcaac

NeuroD2 gacacccccatcctaccc acaggccacccacaggta

SMA catcaccaactgggacga ggtgggatgctcttcagg

SOX17 gcaagatgctgggcaagt ctctgcctcctccacgaa

GATA4 ctggtcttggccgacagt ggtccgtgcaggaatttg

RPL13a cataggaagctgggagcaag gccctccaatcagtcttctg

------------------------------------------------------------------------------------------


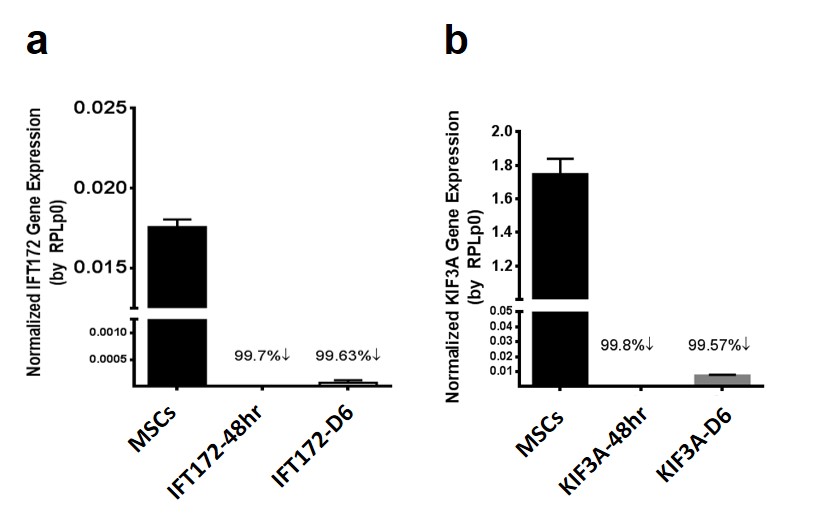


**Fig S1. The efficiency of IFT172 and KIF3A knockdown after 48 hr and on day 6.** The efficiency of siRNA knockdown was validated by quantitative PCR.


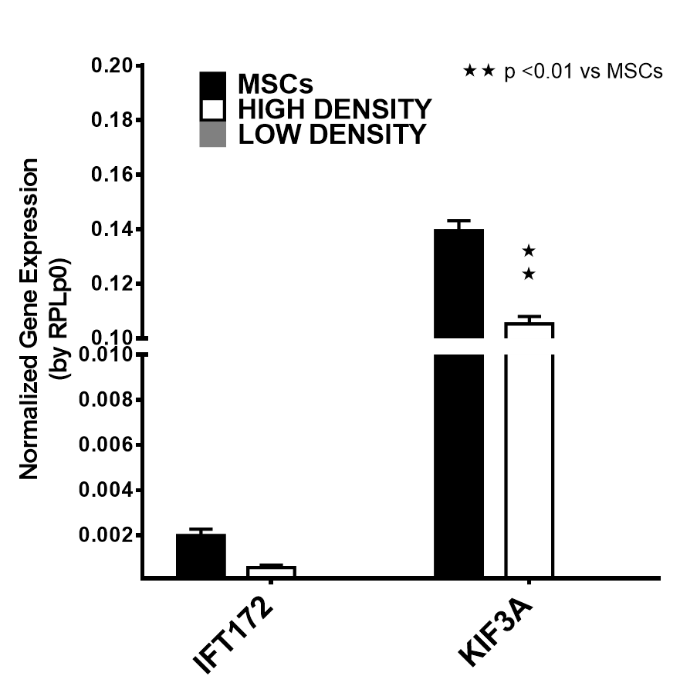


**Fig S2. The efficiency of IFT172 siRNA knockdown.** The efficiency of siRNA knockdown was validated by quantitative PCR
